# Higher-order statistics based multifractal predictability measures for anisotropic turbulence and the theoretical limits of aviation weather forecasting

**DOI:** 10.1038/s41598-019-56304-2

**Published:** 2019-12-27

**Authors:** Arun Ramanathan, A. N. V. Satyanarayana

**Affiliations:** Centre for Oceans, Rivers, Atmosphere and Land sciences (CORAL), Indian Institute of Technology Kharagpur (IIT KGP), Kharagpur, India

**Keywords:** Atmospheric dynamics, Natural hazards, Atmospheric dynamics, Nonlinear phenomena

## Abstract

Theoretical predictability measures of turbulent atmospheric flows are essential in estimating how realistic the current storm-scale strategic forecast skill expectations are. Atmospheric predictability studies in the past have usually neglected intermittency and anisotropy, which are typical features of atmospheric flows, rendering their application to the storm-scale weather regime ineffective. Furthermore, these studies are frequently limited to second-order statistical measures, which do not contain information about the rarer, more severe, and, therefore, more important (from a forecasting and mitigation perspective) weather events. Here we overcome these rather severe limitations by proposing an analytical expression for the theoretical predictability limits of anisotropic multifractal fields based on higher-order autocorrelation functions. The predictability limits are dependent on the order of statistical moment (*q*) and are smaller for larger *q*. Since higher-order statistical measures take into account rarer events, such more extreme phenomena are less predictable. While spatial anisotropy of the fields seems to increase their predictability limits (making them larger than the commonly expected eddy turnover times), the ratio of anisotropic to isotropic predictability limits is independent of *q*. Our results indicate that reliable storm-scale weather forecasting with around 3 to 5 hours lead time is theoretically possible.

## Introduction

Prediction and predictability of the future states of complex systems have always been a significant area of interest in numerous scientific disciplines^1–13^. As far as the field of aviation weather forecasting is concerned, knowledge of the location and intensity of hazardous convective weather about 2 to 6 hours in advance is vital for air traffic planning with minimal weather delays or diversions^14^. While weather forecasts with about two hours lead time are referred to as tactical forecasts, those forecasts with around six hours lead time are known as strategic forecasts by the aviation community. The desired strategic forecasting accuracy may not be achievable using current Numerical Weather Prediction (NWP) techniques^15^, suggesting that instead of some ad-hoc engineered solution, a more fundamental improvement in the understanding of the storm-scale atmospheric predictability limits is vital^16^. Determining the theoretical predictability limits of the storm-scale atmosphere is crucial in knowing if the shortcomings of current strategic mesoscale forecasts are just artifacts of the forecasting techniques used or if we have reached the intrinsic storm-scale atmospheric predictability limit (suggesting that the feat of reliable strategic aviation weather forecasting is theoretically impossible). Earlier works on atmospheric predictability, namely Lorenz’s pioneering chaos theory approach^17^ are not valid in the storm-scale regime as they are unsuitable for systems with a huge number of degrees of freedom^18,19^ whereas the theoretical predictability limits from his seminal scaling approach^20^ neglect intermittency and anisotropy which are typical features of atmospheric flows^21–23^. Although subsequent dynamical systems based studies^24,25^ use much more generalized Lyapunov exponents, recent scaling based predictability studies^19,22,26–29^ neither assume homogeneity nor isotropy of atmospheric fields and incorporate intermittency and anisotropy within generalized emergent scaling laws^30^ that are much more amenable for application in the storm-scale atmosphere. The statistics adopted by earlier atmospheric predictability studies^29^ are both second-order (depending on the square) and also two-point (depending on the separation or lag between two points) that do not take into account the intermittency of the turbulent field nor information about more extreme weather events (this inverse relationship between the statistical order *q* and probability of occurrence of events with fluxes beyond a certain threshold is further explained mathematically in the Results section). Higher-order statistics based predictability measures also need to be considered for overcoming these drawbacks and exploring the whole range of multifractal singularities. In this study an approach based on scaling laws that seem to be ubiquitous in nature^31–34^ is used as explained in the Results section to obtain predictability estimates of the storm-scale atmospheric regime that are discussed in detail followed by a brief conclusion in the Discussion section.

## Results

Since the concepts of both prediction and predictability of complex systems are now widely accepted to be probabilistic^35–40^, we utilize a stochastic Generalized Scale-invariant (GSI) multifractal^27,30,41–44^ based methodology here. Atmospheric space-time scaling laws are of the general form^30^

1 $$\Delta f({\boldsymbol{\Delta }}{\boldsymbol{R}})=|f({\boldsymbol{R}}+\Delta {\boldsymbol{R}})-f({\boldsymbol{R}})|=\varphi \,[\hspace{-2pt}[{\boldsymbol{\Delta }}{\boldsymbol{R}}]\hspace{-2pt}{]}^{H};\varphi =\frac{{\varepsilon }^{\eta }}{\langle {\varepsilon }^{\eta }\rangle }$$

where Δ*f*(**Δ*R***) is the fluctuation of the nonconservative turbulent field *f* across a space-time vector displacement (lag) or scale **Δ*R*** = (Δ*x*, Δ*y*, Δ*z*, Δ*t*), ***R*** = (***r***, *t*) is the space-time position vector, ***r*** = (*x*, *y*, *z*) is the spatial position vector and the angular bracket denotes ensemble averaging. The scaling exponent *H* is the order of fractional integration (*H* > 0) or differentiation (*H* < 0), whereas *φ* is the normalized *η*^th^ power of some conservative turbulent flux field *ε*. In other words, *H* is the conservation or fluctuation exponent, whereas *η* is the exponent of the conservative turbulent flux. The anisotropic space-time scale function [[***R***]] is the general solution of the anisotropic functional scale equation

2 $$\begin{array}{ll}[\kern-2pt[ {T}_{\lambda }{\boldsymbol{R}}]\kern-2pt] ={\lambda }^{-1}[\kern-2pt[ {\boldsymbol{R}}]\kern-2pt] \,; & {T}_{\lambda }={\lambda }^{-{G}_{st}}\\ {G}_{st}=[\begin{array}{cc}{G}_{s} & 0\\ 0 & {H}_{t}\end{array}]; & {G}_{s}=[\begin{array}{ccc}d-c & f+e & 0\\ f-e & d+c & 0\\ 0 & 0 & {H}_{z}\end{array}]\,,\end{array}$$

where *T*_*λ*_ is the scale changing (transformation) operator, *G*_*st*_ and *G*_*s*_ are the space-time and spatial generator matrices, *H*_*t*_ is the dynamic exponent or the space-time anisotropy parameter, *H*_*z*_ is the vertical stratification exponent, *λ* is the scale ratio, and *c,d,e,f* are the generalized scale invariance (GSI) parameters^44^. The spatial elliptical dimension *D*_*el,s*_ equals the trace of the matrix *G*_*s*_, whereas the space-time elliptical dimension *D*_*el,st*_ is the trace of the matrix *G*_*st*_. Following Marsan *et al*.^26,27^ the canonical space-time scale function in real (physical) space can be taken as

3 $$\begin{array}{ccc}[\hspace{-2pt}[(\Delta {\boldsymbol{r}},\Delta t)]\hspace{-2pt}] & = & L({(|\Delta {\boldsymbol{r}}|/L{)}^{2}+{(|\Delta t|/T)}^{2/{H}_{t}})}^{1/2};\\ |\Delta {\boldsymbol{r}}| & = & {(|\Delta x{|}^{2}+|\Delta y{|}^{2}+|\Delta z{|}^{2})}^{1/2},\end{array}$$

where |Δ***r***| is the isotropic spatial scale function, *L* is the integral length scale (usually taken as the size of the largest eddy), and *T* is the eddy turnover time corresponding to *L*. The main difference between real space and Fourier space scale functions is that they are symmetric with respect to different generators: *G*_*s*_ and ${G}_{s}^{{\rm{T}}}$ (this superscript ‘T’ indicates the transpose of a matrix, not to be confused with the integral time scale *T* or the scale transformation operator *T*_*λ*_). For spatially isotropic, self-affine cases or GSI cases with no differential rotation of structures (*e* = 0), *G*_*s*_ is symmetric so that ${G}_{s}={G}_{s}^{{\rm{T}}}$. The Fourier space scale function (indicated by the subscript FS) corresponding to the real space scale function can, therefore, be taken as

4 $$\begin{array}{rcl}{[\kern-2pt[ ({\boldsymbol{k}},\omega )]\kern-2pt] }_{{\rm{FS}}} & = & {K}_{i}{({(|{\boldsymbol{k}}|/{K}_{i})}^{2}+{(|\omega |/{\Omega }_{i})}^{2/{H}_{t}})}^{1/2};\\ |{\boldsymbol{k}}| & = & {(|{k}_{x}{|}^{2}+|{k}_{y}{|}^{2}+|{k}_{z}{|}^{2})}^{1/2};{K}_{i}=\frac{2\pi }{L};{\Omega }_{i}=\frac{2\pi }{T}\,\end{array}$$

where |***k***| is the isotropic spatial scale function in Fourier space, ***k*** = (*k*_*x*_, *k*_*y*_, *k*_*z*_) is the wavevector, *K*_*i*_ and Ω_*i*_ are the (angular) wavenumber and (angular) frequency corresponding to the integral length and time scales (*L* and *T*), respectively. Even though this study prefers the theoretical physics convention of using angular frequency *ω* and angular wavenumber *k* (although the word angular is often dropped in the manuscript for convenience) in the Fourier space instead of the alternate spectroscopy convention of using frequency and spectroscopic wavenumber, both conventions result in the same final outcome as long as they are used consistently. Although real and Fourier space scale functions are equivalent in a scaling sense, they are generally not identical. The canonical scale functions in Eqs. (3 and 4) are simply convenient approximations.

### Semi-fourier space scale functions

Taking into consideration, the scaling anisotropy between space and time (based on the Kolmogorov-Obukhov law^45,46^) suggested by earlier studies^19,47^, $|\omega |\propto {|k|}^{{H}_{t}}$ can be squared on both sides and non-dimensionalized using Ω_*i*_ and *K*_*i*_ to get ${(\frac{|\omega |}{{\Omega }_{i}})}^{2/{H}_{t}}\propto {(\frac{|{\boldsymbol{k}}|}{{K}_{i}})}^{2}.$ Since a spatial-scale dependent but position independent expression for predictability limit is what is needed, it is advantageous to work in semi-Fourier space, fully exploiting this spatial position independence property. The purpose of squaring, is to obtain an expression for ${|\omega |}^{1/{H}_{t}}$ in terms of |Δ*t*|, that can be raised to power 2 and used in Eq. (4) to replace the ${|\omega |}^{\sigma /{H}_{t}}$ term. To do this ${(\frac{|\omega |}{{\Omega }_{i}})}^{2/{H}_{t}}\propto {(\frac{|{\boldsymbol{k}}|}{{K}_{i}})}^{2}$ is rewritten as ${(\frac{|\omega |}{{\Omega }_{i}})}^{\frac{1}{{H}_{t}}}\propto \frac{{(\frac{|{\boldsymbol{k}}|}{{K}_{i}})}^{2}}{{(\frac{|\omega |}{{\Omega }_{i}})}^{\frac{1}{{H}_{t}}}}$, which when using |*ω*|= 2*π*/|Δ*t*| only on the right hand side becomes

5 $${|\omega |}^{1/{H}_{t}}\propto \frac{{({\Omega }_{i})}^{2/{H}_{t}}\,{|\Delta t|}^{1/{H}_{t}}{|{\boldsymbol{k}}|}^{2}}{{(2\pi )}^{1/{H}_{t}}{({K}_{i})}^{2}},$$

which when squared and substituted in Eq. (4) as discussed above gives

6 $${[\kern-2pt[ ({\boldsymbol{k}},\omega )]\kern-2pt] }_{{\rm{FS}}}\propto {({|{\boldsymbol{k}}|}^{2}+\frac{{(L)}^{2}{(\frac{|\Delta t|}{T})}^{\frac{2}{{H}_{t}}}{|{\boldsymbol{k}}|}^{4}}{{(2\pi )}^{2}})}^{\frac{1}{2}}={[\kern-2pt[ ({\boldsymbol{k}},\Delta t)]\kern-2pt] }_{{\rm{SFS}}};$$

where ${K}_{i}=\frac{2\pi }{L};{\Omega }_{i}=\frac{2\pi }{T}$ as usual, and the subscript ‘SFS’ denotes the semi-Fourier space. Using ${E}_{{c}_{q}}({\boldsymbol{k}},\omega )\propto {{[\kern-2pt[ ({\boldsymbol{k}},\omega )]\kern-2pt] }_{FS}}^{-{\beta }_{q}}$ (derived in the Methods section) with Eq. (6) implies that ${E}_{{c}_{q}}({\boldsymbol{k}},\Delta t)\propto {{[\kern-2pt[ ({\boldsymbol{k}},\Delta t)]\kern-2pt] }_{SFS}}^{-{\beta }_{q}}$.

### Theoretical predictability limits of *q*-th order statistical moments

The polyspectrum is defined as a generalized spectrum of order *q* (for *q* = 2, 4, etc. the polyspectrum is the spectrum, tri-spectrum, etc.), and due to statistical stationarity, the total polyspectrum ${E}_{{T}_{q}}({\boldsymbol{k}})={E}_{{c}_{q}}({\boldsymbol{k}},\Delta t)+{E}_{{D}_{q}}({\boldsymbol{k}},\Delta t)$ where ${E}_{{D}_{q}}({\boldsymbol{k}},\Delta t)$ and ${E}_{{c}_{q}}({\boldsymbol{k}},\Delta t)$ are the decorrelated and correlated polyspectra, respectively. At Δ*t* = 0, ${E}_{{D}_{q}}({\boldsymbol{k}},0)=0$ and ${E}_{{C}_{q}}({\boldsymbol{k}},0)={E}_{{T}_{q}}({\boldsymbol{k}})$. Following the pioneering work of Lorenz^20^, that defines the predictability limit as the time until which errors in prediction have not exceeded a prechosen magnitude which for practical purposes should be considerably greater than typical observational errors but less than the magnitude of difference between randomly chosen states of the system, here the theoretical predictability limit Δ*t*_*p*_ is taken as the time when the correlated polyspectrum equals decorrelated polyspectrum (i.e. ${E}_{{D}_{q}}({\boldsymbol{k}},\Delta t)$ is 50% of ${E}_{{T}_{q}}({\boldsymbol{k}})$). Applying the outcome of the Methods section along with Eq. (6) in this definition results in

7 $$\begin{array}{rcl}\frac{{E}_{c}({\boldsymbol{k}},\Delta {t}_{p}(q))}{{E}_{T}({\boldsymbol{k}})} & = & \mu ={(\frac{{[\kern-2pt[ ({\boldsymbol{k}},\Delta {t}_{p}(q))]\kern-2pt] }_{SFS}}{|{\boldsymbol{k}}|})}^{-{\beta }_{q}}\\  & = & {(1+\frac{{(L)}^{2}{(\frac{\Delta {t}_{p}(q)}{T})}^{2/{H}_{t}}{|{\boldsymbol{k}}|}^{2}}{{(2\pi )}^{2}})}^{\frac{-{\beta }_{q}}{2}},\end{array}$$

where $\mu =\frac{1}{2}$ and ${\beta }_{q}=1+qH-K(q\eta )+qK(\eta )$. Equation (7) when solved for Δ*t*_*p*_(*q*) results in the analytical expression

8 $$\Delta {t}_{p}(q)=T{(\frac{{(\mu )}^{\frac{-2}{{\beta }_{q}}}-1}{{(2\pi )}^{-2}{(L)}^{2}{|{\boldsymbol{k}}|}^{2}})}^{\frac{{H}_{t}}{2}}.$$

This predictability limit obtained in a spatially isotropic framework can be directly translated to that of a spatially anisotropic framework by simply replacing the spatially isotropic function |***k***| by a GSI scale function ||***k***||_*FS*_ and the integral length, time scales (*L*,*T*) by the sphero-scale *l*_*s*_ (the spatial scale where structures are roundish), sphero-time *l*_*st*_ (the turnover time corresponding to *l*_*s*_) respectively resulting in the analytical expression for the predictability limit Δ*t*_*p*_(*q*) of the *q*-th order statistical moment of the atmospheric field considered:

9 $$\begin{array}{rcl}\Delta {t}_{p}(q) & = & \frac{{l}_{st}}{{l}_{s}^{{H}_{t}}}{(\frac{{(\mu )}^{\frac{-2}{{\beta }_{q}}}-1}{{(2\pi )}^{-2}{({\Vert {\boldsymbol{k}}\Vert }_{FS})}^{2}})}^{\frac{{H}_{t}}{2}};\\ {\beta }_{q}=1+qH-K(q\eta )+qK(\eta );\,{\Vert {\boldsymbol{k}}\Vert }_{FS} & = & {k}_{s}{({|\frac{{k}_{x}}{{k}_{s}}|}^{2}+{|\frac{{k}_{y}}{a{k}_{s}}|}^{2}+{|\frac{{k}_{z}}{{k}_{s}}|}^{\frac{2}{{H}_{z}}})}^{\frac{1}{2}};\\ {k}_{s} & = & \frac{2\pi }{{l}_{s}};{k}_{st}=\frac{2\pi }{{l}_{st}},\end{array}$$

having linear GSI parameters *c* = *e* = *f* = 0, *d* = 1, horizontal trivial anisotropy parameter *a* and vertical scaling anisotropy parameter *H*_*z*_. The sphero-scale *l*_*s*_ is the scale at which the structures of the field are approximately roundish, the sphero-time *l*_*st*_ is the turnover time of eddies of size *l*_*s*_, whereas *k*_*s*_ and *k*_*st*_ are the sphero-wavenumber and sphero-frequency, respectively. Since $T/{L}^{{H}_{t}}={\bar{\varepsilon }}^{-\frac{1}{3}}\,$($\bar{\varepsilon }$ is the spatially averaged energy flux) and |***k***| is independent of *L*, Eq. (8) is independent of *L*. Equation (9) on the other hand depends on *l*_*s*_ since ||***k***||_*FS*_ depends on *l*_*s*_ (although ${l}_{st}/{l}_{s}^{{H}_{t}}={\bar{\varepsilon }}^{-\frac{1}{3}}$). The critical ratio *μ* of the correlated polyspectra to that of the total polyspectra (the polyspectrum is a generalized spectrum of order *q* as described earlier) specifies how much error in prediction is acceptable, *a* and *H*_*z*_ are the trivial (non-scaling) horizontal and scaling vertical anisotropy parameters respectively, whereas *H*_*t*_ is the space-time anisotropy parameter. The scaling moment function *K*(*q*) is given as $\frac{{C}_{1}}{\alpha -1}({q}^{\alpha }-q)$, where *C*_1_ is the codimension of the mean, and *α* is the index of multifractality. The basic cascade equation for a scale by scale conserved multifractal field is given as: $\langle {\varepsilon }_{\lambda }^{q}\rangle ={\lambda }^{K(q)}$, where the angular brackets denote ensemble averaging, *q* the order of the statistical moment, and the scale ratio *λ*= Largest scale/intermediate scale. The scaling moment function *K*(*q*) that describes how the statistical properties of each moment behave under scale transformations^48^ is also the (base *λ*, Laplace) second characteristic function (SCF). The smoothness parameter *H* is used to obtain non-conservative observed fields from conservative multifractal cascade processes, whereas *η* is the exponent of the conservative turbulent flux (not to be confused with the Kolmogorov scale usually denoted by *η* in turbulence literature). The significance of *η* is that for observed non-conservative fields such as the velocity shear across a scale *l*, based on the physical notion of eddy turnover time or purely dimensional considerations are directly proportional to the *η*-th power of the conservative fields such as the energy flux density (for the case of horizontal wind shear *η* = 1/3). Spatial GSI scale functions are denoted by || ||, and in Fourier space (*k*_*x*_, *k*_*y*_, *k*_*z*_) as ||***k***||_*FS*_, where the wave vector ***k*** = (*k*_*x*_, *k*_*y*_, *k*_*z*_) has the Euclidean norm $|{\boldsymbol{k}}|={(|{k}_{x}{|}^{2}+|{k}_{y}{|}^{2}+|{k}_{z}{|}^{2})}^{1/2}$.

### Empirical-parameter based estimate of predictability limits

**Figure 1:**
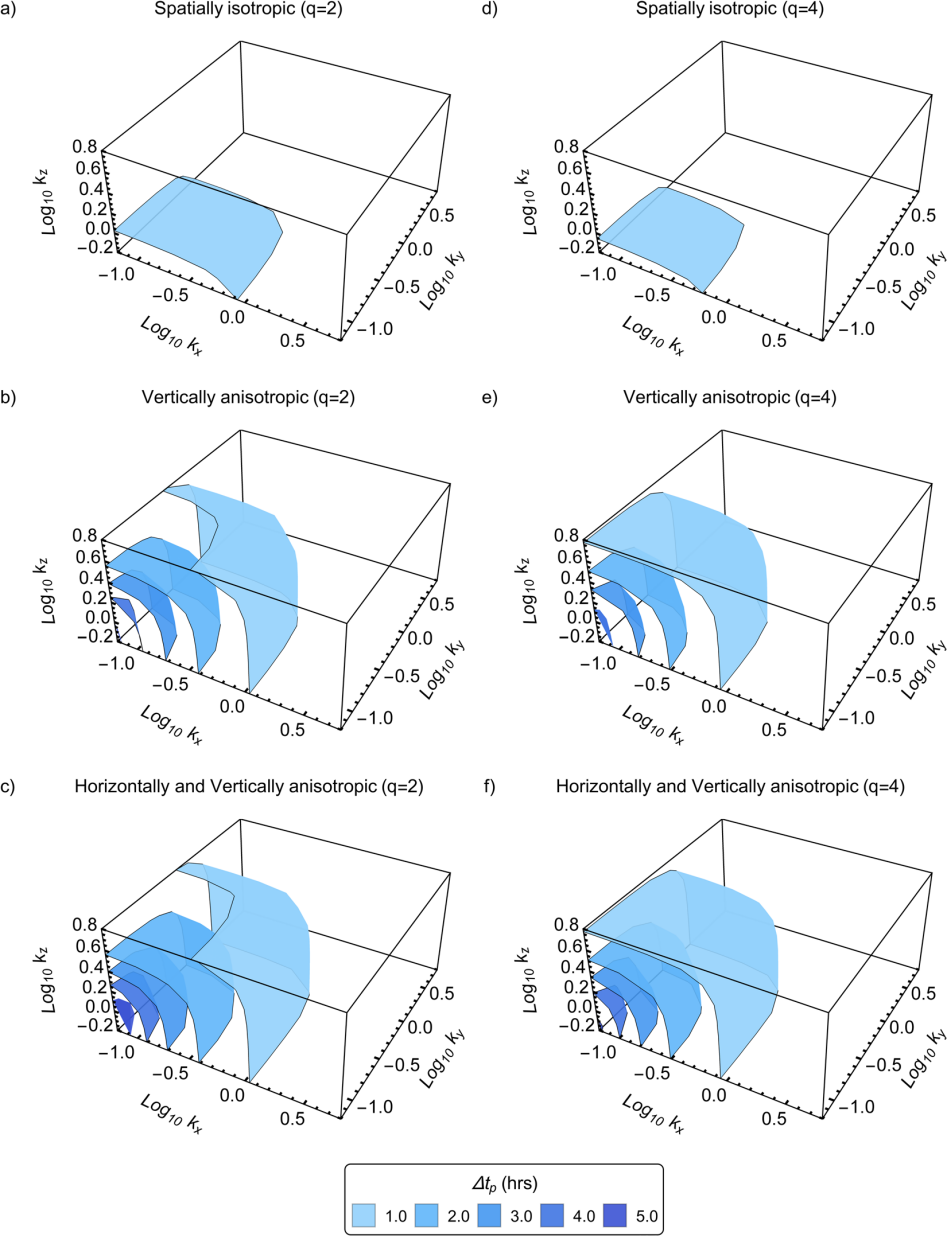
Theoretical predictability limits of spatially isotropic and anisotropic (with 100 *m* sphero-scale) horizontal wind fields. The wind fields have multifractal parameters $\alpha =1.5,{C}_{1}=0.15,H=0.33,$ and anisotropy parameters as discussed in the text. The wavenumbers *k*_*x*_, *k*_*y*_, *k*_*z*_ have units of km^−1^ and are in the *x*, *y*, *z* directions respectively, whereas the predictability limits are in hours. The horizontal wavenumbers represent a scale range from 1 to 100 km, whereas the vertical wavenumber represents a scale range from 1 to 10 km (scales smaller than 1 km are not shown here as their predictability limits are less than 1 hour). The two rows differ only by the order of autocorrelation *q* used in deriving the predictability limits. (**a)** spectra based predictability limits (*q* = 2) of an isotropic horizontal wind field as a function of logarithmic wavenumber. (**b)** same as **a**, but for vertically anisotropic cases. (**c)** same as **a**, but for horizontally and vertically anisotropic cases. (**d–f)** are same as (**a**–**c**) but for *q* = 4. Comparing figures (**a–c**) with (**d**–**f)** shows that higher-order predictability limits are smaller than lower-order ones. Since higher-order statistical moments represent more extreme events, these figures indicate that such events are less predictable. Comparing figures (**a**–**c)** with each other and figures (**d**–**f**) with each other illustrates that fields that are more anisotropic are more predictable.

**Figure 2:**
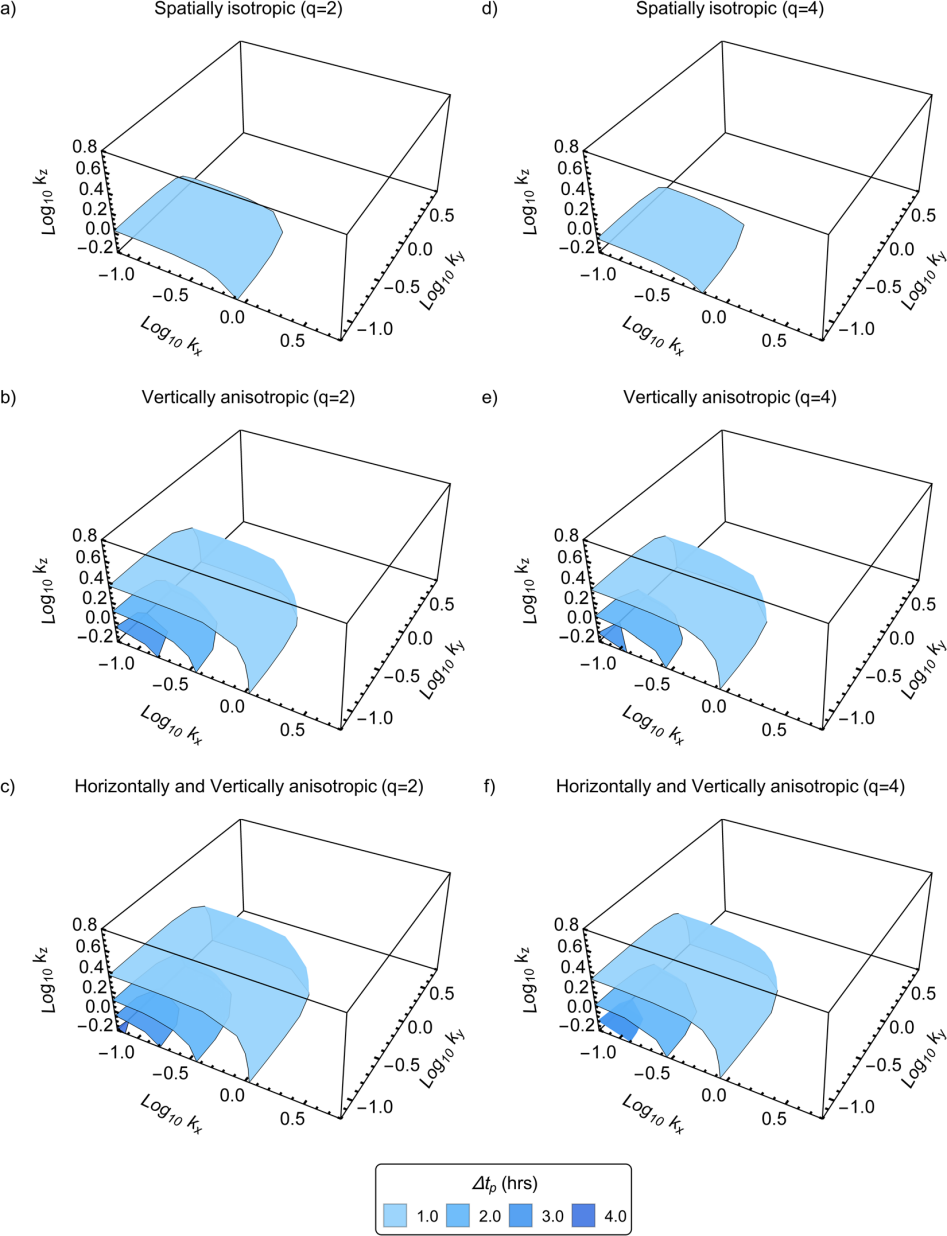
Theoretical predictability limits of spatially isotropic and anisotropic (with 1000 *m* sphero-scale) horizontal wind fields. The wind fields have multifractal parameters $\alpha =1.5,{C}_{1}=0.15,H=0.33,$ and anisotropy parameters as discussed in the text. The wavenumbers *k*_*x*_, *k*_*y*_, *k*_*z*_ have units of km^−1^ and are in the *x*, *y*, *z* directions respectively, whereas the predictability limits are in hours. The horizontal wavenumbers represent a scale range from 1 to 100 km, whereas the vertical wavenumber represents a scale range from 1 to 10 km (scales smaller than 1 km are not shown here as their predictability limits are less than 1 hour). The two rows differ only by the order of autocorrelation *q* used in deriving the predictability limits. (**a**) spectra based predictability limits (*q* = 2) of an isotropic horizontal wind field as a function of logarithmic wavenumber. (**b**) same as a, but for vertically anisotropic cases. (**c**) same as a, but for horizontally and vertically anisotropic cases. (**d–f**) are same as a, b, c but for *q* = 4. Comparing figures (**a–c**) with (**d–f**) shows that higher-order predictability limits are smaller than lower-order ones. Since higher-order statistical moments represent more extreme events, these figures indicate that such events are less predictable. Comparing figures (**a–c**) with each other and figures (**d–f**) with each other illustrates that fields that are more anisotropic are more predictable. Comparing Fig. 1 with this figure shows that fields with larger sphero-scales are less predictable.

Empirical estimates of multifractal parameters *α* = 1.5,*C*_1_ = 0.15 used by earlier works^26^ and $H=0.33,\eta =1/3,{H}_{t}=2/3$ suggested by the Kolmogorov-Obukhov law^45,46^ are used along with horizontal, vertical anisotropy parameters *a* = 1.6 (an ECMWF interim flux based estimate^49^), *H*_*z*_ = 5/9 (following Schertzer and Lovejoy^50^), a typical storm-scale sphero-scale of 100 *m* (determined from CloudSat data by Lovejoy *et al*.^51^), *μ* = 0.5 (following the critical ratio used by Schertzer and Lovejoy^19,22^) in Eq. (9) for assessing the predictability limits of horizontal wind fields. In this study, the isotropic wind fields have *a* = 1, *H*_*z*_ = 1; horizontally anisotropic fields have *a* = 1.6, *H*_*z*_ = 1; whereas the horizontally and vertically anisotropic wind fields have *a* = 1.6, *H*_*z*_ = 5/9. For such horizontally and vertically anisotropic wind fields in the convective regime which is typically 100 *km* in the horizontal^30^ and 10 *km* in the vertical, the maximum predictability limits occur at the largest scales of the regime (100 *km*,100 *km*,10 *km*) as can be inferred from Eq. (9) and are about 5 *hrs* and 4 *hrs* for *q* = 2 and *q* = 4 respectively. The limits derived using larger *q* values are smaller as expected (as illustrated by Fig. 1), since rarer events are less predictable^19,22^. Fields with a larger sphero-scale of 1000 *m* have predictability limits that are smaller (the maximum values are about 4*hrs* and 3*hrs* for *q* = 2 and *q* = 4 respectively) than those of fields with 100 *m* spheroscale, as anticipated (as illustrated by Fig. 2). This is a consequence of the sub- and super-spheroscales being dominated by the buoyancy variance flux and energy flux respectively^50^, and systems with stratiform dynamics having lesser buoyancy variance flux than those with convective dynamics^30^. Finally, these figures also show that anisotropy improves predictability in accordance with earlier spectra based assessments.

### Probability of occurrence

The scaling moment function, *K*(*q*) is related to the codimension of the order of singularities, $c(\gamma )={C}_{1}{(\frac{\gamma (\alpha -1)}{\alpha {C}_{1}}+\frac{1}{\alpha })}^{\frac{\alpha }{\alpha -1}}$ via the Legendre transform^52^, $K(q)=\mathop{\max }\limits_{\gamma }(q\gamma -c(\gamma ))$. The *γ* that maximizes *qγ* − *c*(*γ*) is denoted by *γ*_*q*_ and is the solution of the equation *c*′(*γ*_*q*_) = *q*, where *c*′(*γ*) is the first derivative of the codimension of order of singularities in *γ*. For *q* = 2, 4 the corresponding *γ*_2_,*γ*_4_ are computed using this equation with empirical estimates of *α*,*C*_1_ (as discussed earlier). By substituting these order of singularity values, the corresponding codimension of order of singularities are then computed. The probability of occurrence of events above a scaling threshold^30^, ${\rm{\Pr }}({{\boldsymbol{\varepsilon }}}_{{\boldsymbol{\lambda }}}\ge {\lambda }^{{{\boldsymbol{\gamma }}}_{{\boldsymbol{q}}}}) \sim {\lambda }^{-{\boldsymbol{c}}({{\boldsymbol{\gamma }}}_{{\boldsymbol{q}}})}$ is then obtained for different scales but with the largest scale being 10000 *km* (since in the atmosphere, the scales typically vary from 1*mm* to 10000 *km*). A straightforward mathematical simplification of the above discussion shows that $c({\gamma }_{q})={C}_{1}{q}^{\alpha }$ (where both *α* and *C*_1_ are positive), implying that larger the statistical order *q* larger the codimension of singularities $c({\gamma }_{q})$ corresponding to that order and therefore smaller the probability of occurrence of flux events with order of singularities exceeding *γ*_*q*_. This means that higher-order statistical moments are more representative of less probable or extreme events.

## Discussion

**Figure 3:**
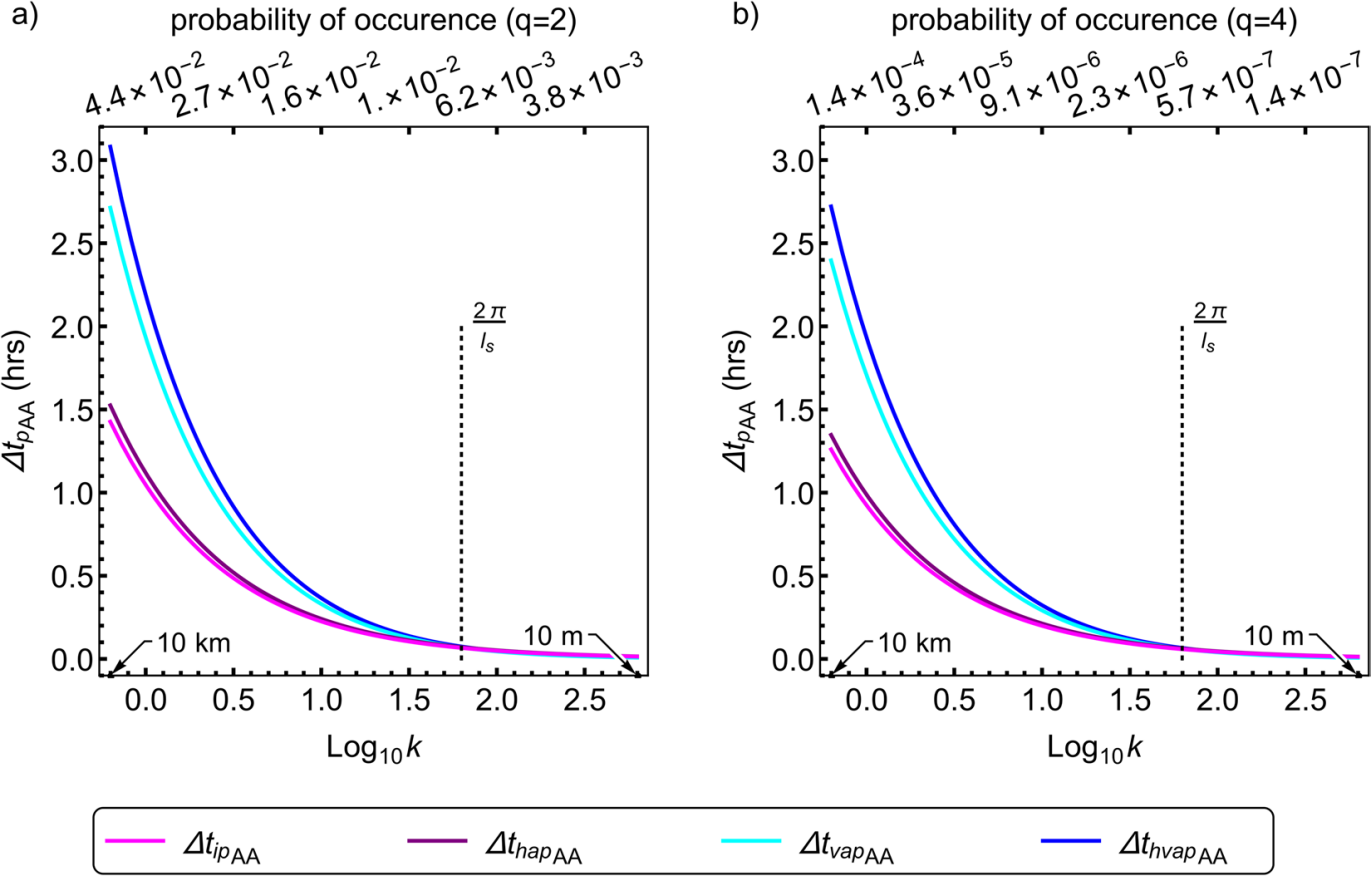
Angular averaged theoretical predictability limits of spatially isotropic and anisotropic (with 100 *m*sphero-scale) horizontal wind fields. The wind field has multifractal parameters $\alpha =1.5,{C}_{1}=0.15,H=0.33,$ and anisotropy parameters, as discussed in the text. The subscripts *ip*,*hap*,*vap*,*hvap* denote isotropic, horizontally anisotropic, vertically anisotropic, horizontally and vertically anisotropic cases, whereas the subscript *AA* indicates angular averaging. While the angular averaged predictability limits is in hours, the wavenumber *k* is in km^−1^ and corresponds to scales ranging from 10 m to 10 km. The two panels differ only by the order of autocorrelation *q* used in deriving the predictability limits. (**a**) spectra based angular averaged predictability limits (*q* = 2) as a function of logarithmic wavenumber. The probability of occurrence shows the probability distribution of energy fluxes above the scaling threshold (${\lambda }^{{\gamma }_{q}}$, where $\lambda =\frac{{\rm{Largest}}\,{\rm{scale}}}{{\rm{Intermediate}}\,{\rm{scale}}\,}=\frac{10,000\,{\rm{km}}}{l}=\frac{10,000\,{\rm{km}}}{|\Delta {\boldsymbol{r}}|}$ is the scale ratio, *l* ranges from 0.01 km to 10 km, and *γ*_*q*_ is the order of singularity corresponding to the order of moment *q*) given by ${\rm{\Pr }}({\varepsilon }_{\lambda }\ge {\lambda }^{{\gamma }_{q}}) \sim {\lambda }^{-c({\gamma }_{q})}$ (where *c*(*γ*) is the codimension of the order of singularities and is related to *K*(*q*) as shown in Sect 4.3), corresponding to wavenumbers 10^0^,10^0.5^,10^1.0^,10^1.5^,10^2.0^,10^2.5^ (see text). The wavenumber $k=|{\boldsymbol{k}}|=\frac{2{\rm{\pi }}}{l}$ (*l* ranges from 0.01 km to 10 km). In other words $\lambda \propto \frac{1}{l}\propto k$ (although *λ* and *k* are not exactly equal in these figures, they are both related through *l*). (**b**) same as a, but for polyspectra based angular averaged predictability limits (*q* = 4). The curves in both figures (**a,b**) show that fields which are more anisotropic are more predictable (over scales larger than the sphero-scale *l*_*s*_) and that larger events are more probable and predictable, whereas comparison between these two figures indicates that more extreme events (less probable) are less predictable.

**Figure 4:**
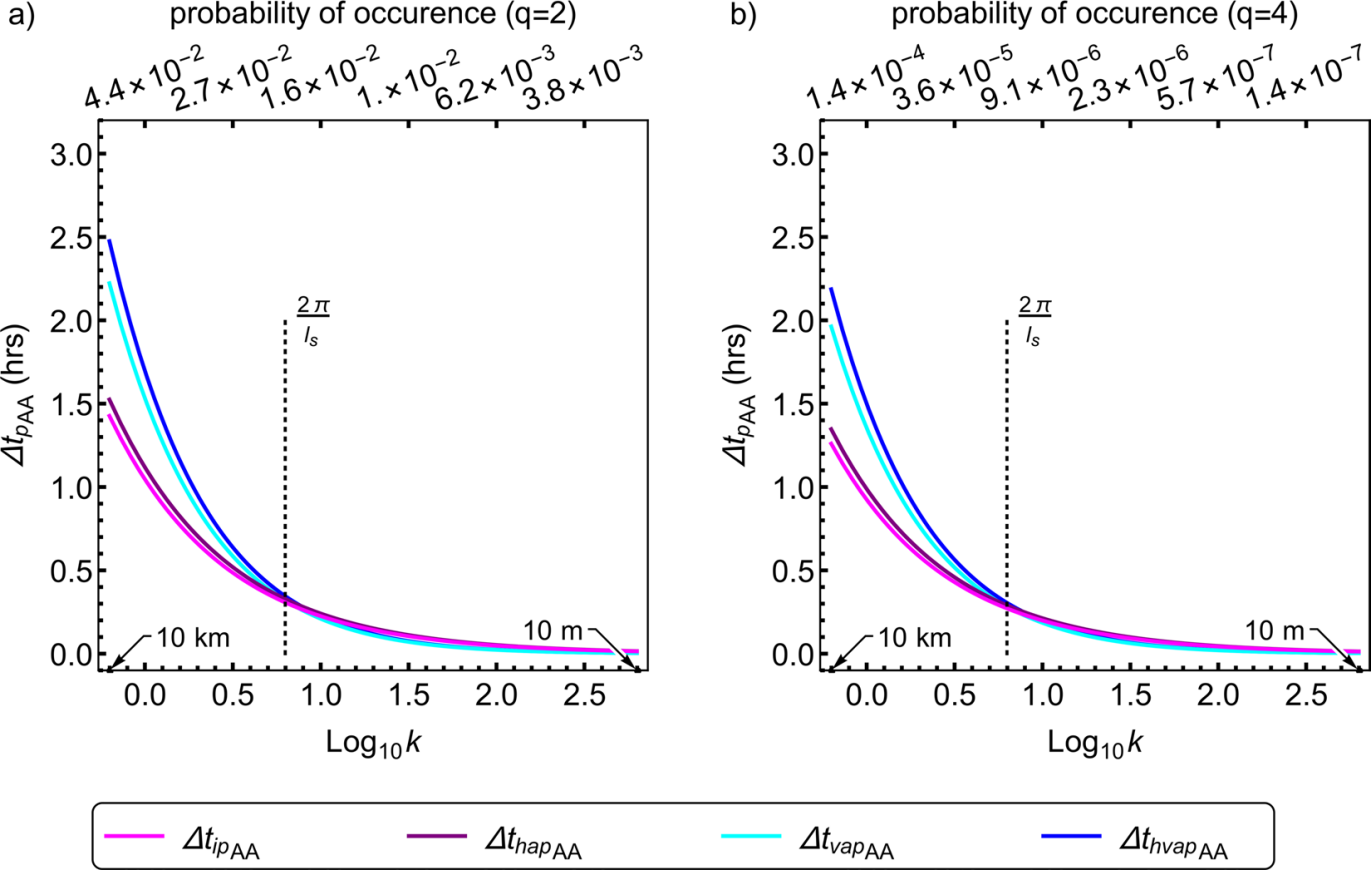
Angular averaged theoretical predictability limits of spatially isotropic and anisotropic (with 1000 *m* sphero-scale) horizontal wind fields. The wind field has multifractal parameters $\alpha =1.5,{C}_{1}=0.15,H=0.33,$ and anisotropy parameters, as discussed in the text. The subscripts *ip*,*hap*,*vap*,*hvap* denote isotropic, horizontally anisotropic, vertically anisotropic, horizontally and vertically anisotropic cases, whereas the subscript *AA* indicates angular averaging. While the angular averaged predictability limits is in hours, the wavenumber *k* is in km^−1^ and corresponds to scales ranging from 10 m to 10 km. The two panels differ only by the order of autocorrelation *q* used in deriving the predictability limits. (**a**) spectra based angular averaged predictability limits (*q* = 2) as a function of logarithmic wavenumber. The probability of occurrence shows the probability distribution of energy fluxes above the scaling threshold (${\lambda }^{{\gamma }_{q}}$, where $\lambda =\frac{{\rm{Largest}}\,{\rm{scale}}}{{\rm{Intermediate}}\,{\rm{scale}}\,}=\frac{10,000\,{\rm{km}}}{l}=\frac{10,000\,{\rm{km}}}{|\Delta {\boldsymbol{r}}|}$ is the scale ratio, *l* ranges from 0.01 km to 10 km, and *γ*_*q*_ is the order of singularity corresponding to the order of moment *q*) given by ${\rm{\Pr }}({\varepsilon }_{\lambda }\ge {\lambda }^{{\gamma }_{q}}) \sim {\lambda }^{-c({\gamma }_{q})}$ (where *c*(*γ*) is the codimension of the order of singularities and is related to *K*(*q*) as shown in Sect 4.3), corresponding to wavenumbers 10^0^,10^0.5^,10^1.0^,10^1.5^,10^2.0^,10^2.5^ (see text). The wavenumber $k=|{\boldsymbol{k}}|=\frac{2{\rm{\pi }}}{l}$ (*l* ranges from 0.01 km to 10 km). In other words $\lambda \propto \frac{1}{l}\propto k$ (although *λ* and *k* are not exactly equal in these figures, they are both related through *l*). (**b**) same as a, but for polyspectra based angular averaged predictability limits (*q* = 4). The curves in both figures (**a,b**) show that fields which are more anisotropic are more predictable (over scales larger than the sphero-scale *l*_*s*_) and that larger events are more probable and predictable, whereas comparison between these two figures indicates that more extreme events (less probable) are less predictable. Comparing Fig. 3 with this figure shows that fields with smaller sphero-scales are more predictable.

Figures 1 and 2, although informative do not directly show how the predictability limits are affected due to anisotropy at super-spheroscales (scales larger than the sphero-scale) and sub-spheroscales (scales smaller than the spheroscale). Since this sphero-scale *l*_*s*_ is the same in all three directional planes, it is independent of the direction. Therefore, it is necessary to get the predictability limit into a similar direction independent format. To do this Δ*t*_*p*_ has to become independent of both the azimuthal and polar angles, and angular averaging seems to be the simplest way of doing this in a more generalized manner (this loss of directional information along with the scale being limited by the smallest of the three scales are the drawbacks of doing this). Angular averaging Eq. (9) for further investigation, results in the angular averaged predictability limit [Δ*t*_*p*_(*q*)]_*AA*_ given by

10 $$\begin{array}{rcl}{[\Delta {t}_{p}(q)]}_{AA} & = & \frac{{l}_{st}}{{l}_{s}^{{H}_{t}}}{({(\mu )}^{\frac{-2}{{\beta }_{q}}}-1)}^{\frac{{H}_{t}}{2}}(\frac{2}{\pi }\,\frac{2}{\pi })\underset{0}{\overset{\frac{\pi }{2}}{\int }}\underset{0}{\overset{\frac{\pi }{2}}{\int }}{(\frac{{\Vert {\boldsymbol{k}}\Vert }_{FS}}{2\pi })}^{-{H}_{t}}d\theta d\phi ;\\ {\Vert {\boldsymbol{k}}\Vert }_{FS} & = & \frac{2\pi }{{l}_{s}}{({(\frac{{l}_{s}}{2\pi })}^{2}{k}_{x}^{2}+{(\frac{{l}_{s}}{2\pi a})}^{2}{k}_{y}^{2}+{(\frac{{l}_{s}}{2\pi })}^{\frac{2}{{H}_{z}}}{k}_{z}^{\frac{2}{{H}_{z}}})}^{\frac{1}{2}};\\ k & = & |{\boldsymbol{k}}|={({k}_{x}^{2}+{k}_{y}^{2}+{k}_{z}^{2})}^{1/2};{k}_{x}=|{\boldsymbol{k}}|\sin \,\phi \,\cos \,\theta ;\\ {k}_{y} & = & |{\boldsymbol{k}}|\sin \,\phi \,\sin \,\theta ;{k}_{z}=|{\boldsymbol{k}}|\cos \,\phi ;0\le \theta \le \frac{\pi }{2};0\le \phi \le \frac{\pi }{2}\end{array}$$

where *θ* and *ϕ* are the polar and azimuthal angles, respectively. It is sufficient to consider only non-negative wavenumbers *k*_*x*_, *k*_*y*_ and *k*_*z*_ since the Fourier space scale function ||***k***||_*FS*_ we use is an even function, as can be seen from Eq. (9), due to which the results are symmetric in the negative part. Therefore, we consider only the first quadrant $(0,\frac{\pi }{2})$ where (sin*ϕ* cos*θ*), (sin*ϕ* sin*θ*) and (cos*ϕ*) are all non-negative. Figure 3a) shows the spectra based angular averaged predictability limits (*q* = 2) of a horizontal wind field with *l*_*s*_ = 100 *m*, as a function of logarithmic wavenumber. The probability of occurrence shows the probability distribution of energy fluxes (*ε*) above the scaling threshold^30^ (*λ*^*γ*^, where $\lambda =\frac{{\rm{Largest}}\,{\rm{scale}}}{{\rm{Intermediate}}\,{\rm{scale}}}$ is the scale ratio and *γ* is the order of singularity) given by ${\rm{\Pr }}({\varepsilon }_{\lambda }\ge {\lambda }^{\gamma }) \sim {\lambda }^{-c(\gamma )}$ (where *c*(*γ*) is the codimension of the order of singularities and is related to *K*(*q*)), corresponding to wavenumbers 10^0^,10^0.5^,10^1.0^,10^1.5^,10^2.0^,10^2.5^. The angular averaged predictability limits of the isotropic, horizontally isotropic, vertically anisotropic, horizontally and vertically anisotropic wind fields are denoted by ${[\Delta {t}_{ip}]}_{AA},{[\Delta {t}_{hap}]}_{AA},{[\Delta {t}_{vap}]}_{AA},{[\Delta {t}_{hvap}]}_{AA}$, respectively. At scales around 10 *km*, about 5% of the energy fluxes contribute to the second-order statistical moment, and the horizontally and vertically anisotropic horizontal wind field corresponding to these fluxes has an angular averaged predictability limit of 3.1*hrs*. Figure 3b) is the same as 3a), but for *q* = 4. The scale ratio $(\lambda =\frac{L}{l}=\frac{L}{|\Delta {\boldsymbol{r}}|})$ is the ratio of the largest scale *L* (10,000 km) to the intermediate scale *l* (ranging from 10 m to 10 km), whereas the wavenumber $k=|{\boldsymbol{k}}|=\frac{2{\rm{\pi }}}{l}$ (here also *l* ranges from 10 m to 10 km). In other words $\lambda \propto \frac{1}{l}\propto k$ (although they are not exactly equal in these figures, they are both related through *l*). At scales around 10 *km*, about 0.025% of the energy fluxes contribute to the fourth-order statistical moment, and the horizontally and vertically anisotropic horizontal wind field corresponding to these fluxes has an angular averaged predictability limit of 2.7 *hrs*. Vertical stratification seems to improve predictability only in the subsphero-wavenumbers. Figure 4 is the same as Fig. 3 but is for a 1000 *m* sphero-scale. At scales around 10 *km*, about 5% of the energy fluxes contribute to the second-order statistical moment, and the horizontally and vertically anisotropic horizontal wind field corresponding to these fluxes has an angular averaged predictability limit of 2.5 *hrs*. Figure 4b) same as 4a), but for *q* = 4. At scales around 10 *km*, about 0.025% of the energy fluxes contribute to the fourth-order statistical moment, and the horizontally and vertically anisotropic horizontal wind field corresponding to these fluxes has an angular averaged predictability limit of 2.2 *hrs*.

**Figure 5:**
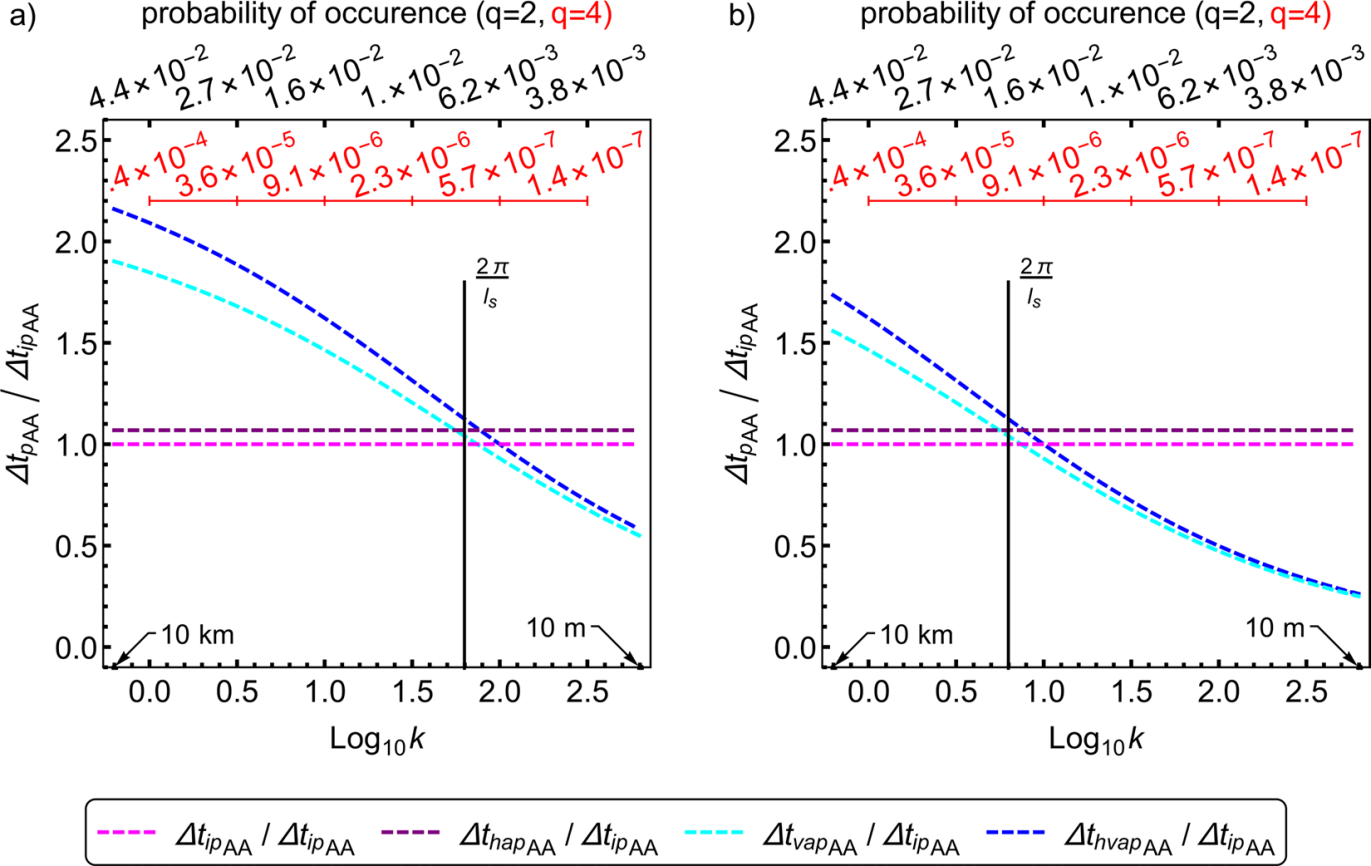
Ratio of the angular averaged theoretical predictability limits of anisotropic horizontal wind fields to that of isotropic horizontal wind fields. The wind fields have multifractal parameters $\alpha =1.5,{C}_{1}=0.15,H=0.33,$ and anisotropy parameters, as discussed in the text. The two panels differ only by the sphero-scale *l*_*s*_. The subscripts *ip*,*hap*,*vap*,*hvap* denote isotropic, horizontally anisotropic, vertically anisotropic, horizontally and vertically anisotropic cases, whereas the subscript *AA* indicates angular averaging. The wavenumber *k* = |***k***| is in km^−1^ and corresponds to scales ranging from 10 m to 10 km. The ratios of the angular averaged predictability limits are independent of the order of statistical moment *q* and, therefore, of how probable the occurrence of the event is. (**a**) the spatially anisotropic fields have *l*_*s*_ = 100 *m*. (**b**) same as a, but the spatially anisotropic fields have *l*_*s*_ = 1000 *m*. The probability of occurrence values in Fig. (**a,b**) are the same as those in Figs. 3 and 4. Vertical stratification enhances and diminishes predictability in the subsphero and supersphero-wavenumbers. Horizontal stratification improves predictability over all scales. Wind fields with smaller spheroscales are better predictable. Larger events are more probable and have larger anisotropic to isotropic angular averaged predictability limit ratios.

Finally, Fig. 5 shows the importance of sphero-scales. The two panels differ only by the sphero-scale *l*_*s*_, and the ratios of the angular averaged predictability limits are independent of the order of statistical moment *q*. In Fig. 5a) *l*_*s*_ = 100 *m*, at scales around 10 *km*, about 5% and 0.025% of the energy fluxes contribute to the second and fourth-order statistical moments and the horizontal wind field corresponding to these fluxes when subject to both horizontally and vertically anisotropic has an angular averaged predictability limit that is 2.15 times the isotropic limit. Figure 5b) is the same as a), but has *l*_*s*_ = 1000 *m*. At scales around 10 *km*, about 5% and 0.025% of the energy fluxes contribute to the second and fourth-order statistical moments and the horizontal wind field corresponding to these fluxes when subject to both horizontally and vertically anisotropic has an angular averaged predictability limit that is 1.75 times the isotropic limit. Vertical stratification enhances and diminishes predictability in the subsphero and supersphero-wavenumbers. Horizontal stratification improves predictability over all scales, although the improvement is not very significant. Figure 5a,b) show that wind fields with smaller spheroscales are better predictable as they are less dominated by convective dynamics.

In conclusion, (i) the super and subsphero-scale predictability is enhanced and diminished correspondingly for scaling anisotropy, (ii) for trivial anisotropy, predictability over the entire scale range is improved in accordance with spectra based estimates, (iii) reliably forecasting convectively less dominant systems that are more probable to occur with around 5 hours lead time seems to be theoretically possible (in case of less probable events this lead time is reduced to 4 hours), iv) whereas reliably forecasting convectively more active systems that are more probable to occur with around 4 hours lead time seems to be theoretically possible (in case of less probable events this lead time is reduced to 3 hours). Although convective scale numerical models are capable of reliably forecasting events with stronger large-scale forcing (organized convection) sometimes even out to 4 hours (around the theoretical limit proposed for convectively active and more probable events) in some cases (when initialized with high-resolution Doppler radar observations), their ability to predict air-mass type storms (unorganized convection) is still quite low^53–56^ (not even close to the theoretical limit proposed here for convectively active and more extreme or less probable events). Even recent predictability studies^57,58^ using storm-scale ensemble forecasting systems conclude that these convective-allowing sophisticated NWP models perform poorly (the predictability of scales smaller than 100 *km* is totally lost at around 1*hr* - which is quite low compared to the theoretical limits proposed in this present study) especially for quantitative precipitation forecasting, and that further effort is therefore needed in improving the basic understanding storm-scale weather predictability. The results of this study, demonstrates that the current expectations of reliable aviation weather forecasts with 2 to 6 *hrs* lead times (i.e., strategic aviation weather forecasting) are not totally unrealistic subject to the incorporation of multifractal cascade dynamics based modeling strategies (especially for unorganized convective weather phenomena which are difficult to forecast using conventional convective scale NWP models).

## Methods

### Estimation of the scaling exponent of correlation polyspectra

Following earlier studies^27,29^ and using Eq. (1), the *q*-th order structure function, for even integer *q* can be written using the binomial theorem as

11 $$\begin{array}{rcl}\langle {(\Delta f(\Delta {\boldsymbol{R}}))}^{q}\rangle  & = & \langle {(f({\boldsymbol{R}}+\Delta {\boldsymbol{R}})-f({\boldsymbol{R}}))}^{q}\rangle \\  & = & \mathop{\sum }\limits_{n=0}^{q}{(-1)}^{n}(\begin{array}{c}q\\ n\end{array})\langle {(f({\boldsymbol{R}}+\Delta {\boldsymbol{R}}))}^{q-n}{(f({\boldsymbol{R}}))}^{n}\rangle ,\end{array}$$

due to statistical translational invariance (the terms (***R*** + Δ***R***) and ***R*** are interchangeable in $\langle {(f({\boldsymbol{R}}+\Delta {\boldsymbol{R}}))}^{q-n}{(f({\boldsymbol{R}}))}^{n}\rangle$). The space-time vector lag ***ΔR*** = (Δ*x*, Δ*y*, Δ*z*, Δ*t*), and the angular brackets indicate ensemble averaging, whereas $$(\begin{array}{c}q\\ n\end{array})=\frac{q!}{(q-n)!n!}$$. Equation (11) implies that the total statistical moment of order *q* (i.e. $\langle {(f({\boldsymbol{R}}+\Delta {\boldsymbol{R}}))}^{q}\rangle +\langle {(f({\boldsymbol{R}}))}^{q}\rangle$) equals the sum of the decorrelated (i.e. $\langle {(\Delta f(\Delta {\boldsymbol{R}}))}^{q}\rangle$) and correlated (i.e. $${\sum }_{n=1}^{q-1}{(-1)}^{n+1}(\begin{array}{c}q\\ n\end{array})\langle {(f({\boldsymbol{R}}+\Delta {\boldsymbol{R}}))}^{q-n}{(f({\boldsymbol{R}}))}^{n}\rangle $$) moments of order *q*. As per the (*q* − 1)*d*-dimensional Wiener-Khinchin theorem^59,60^, the generalized (*q* − 1)th order autocorrelation function $u({\boldsymbol{\Delta }}{{\boldsymbol{R}}}_{1},\ldots ,{\boldsymbol{\Delta }}{{\boldsymbol{R}}}_{q-1})$ and the corresponding generalized correlation polyspectral ((*q* − 1)th order) density^61^ ${P}_{{c}_{q}}({{\boldsymbol{K}}}_{1},\ldots ,{{\boldsymbol{K}}}_{q-1})$ (the subscript *c*_*q*_ of the polyspectral density means that it is the correlated polyspectral density and is dependent on the order of the statistical moment *q*, but it does not mean that it is of the *q*-th order) are related to each other via the (*q* − 1)*d* dimensional inverse Fourier transform

12 $$\begin{array}{rcl}u({\boldsymbol{\Delta }}{{\boldsymbol{R}}}_{1},\ldots ,{\boldsymbol{\Delta }}{{\boldsymbol{R}}}_{q-1}) & = & \langle f({\boldsymbol{R}})f({\boldsymbol{R}}+\Delta {{\boldsymbol{R}}}_{1})\ldots f({\boldsymbol{R}}+\Delta {{\boldsymbol{R}}}_{q-1})\rangle \\  & = & \int \ldots {\int }_{{D}^{(q-1)d}}{e}^{i({K}_{1}\Delta {{\boldsymbol{R}}}_{1}+\ldots +{{\boldsymbol{K}}}_{q-1}\Delta {{\boldsymbol{R}}}_{q-1})}{P}_{{c}_{q}}({{\boldsymbol{K}}}_{1},\mathrm{.}.,{{\boldsymbol{K}}}_{q-1})\,{d}^{d}{{\boldsymbol{K}}}_{1}\ldots {d}^{d}{{\boldsymbol{K}}}_{q-1}\end{array}$$

where the whole domain of integration is over the region in *D*^(*q*−1)*d*^ (since here we deal with three-dimensional space and one-dimensional time, *d* = 4) as there are (*q* − 1) of these *d*-dimensional integrals. Due to the assumption of statistical translational invariance the right-hand side is independent of ***R*** and is dependent only on the lags ${\boldsymbol{\Delta }}{{\boldsymbol{R}}}_{1},\ldots ,{\boldsymbol{\Delta }}{{\boldsymbol{R}}}_{q-1}$. When (*q* − *n*) of these (*q* − 1) lags are equal to **Δ*R***, and the remaining (*n* − 1) lags are zero Eq. (12) becomes

13 $$\langle {(f({\boldsymbol{R}}+\Delta {\boldsymbol{R}}))}^{q-n}{(f({\boldsymbol{R}}))}^{n}\rangle =\int \ldots {\int }_{{D}^{(q-1)d}}{e}^{i(q-n)({\boldsymbol{K}}\Delta {\boldsymbol{R}})}{P}_{{c}_{q}}({\boldsymbol{K}}){d}^{d}{\boldsymbol{K}}\ldots {d}^{d}{\boldsymbol{K}}$$

Using Eq. (13) in the binomial expansion given by Eq. (11) results in

14 $$\langle {(\Delta f(\Delta {\boldsymbol{R}}))}^{q}\rangle =\int \ldots {\int }_{{D}^{(q-1)d}}(\mathop{\sum }\limits_{n-0}^{q}{(-1)}^{n}(\begin{array}{c}q\\ n\end{array}){e}^{i(q-n)({\boldsymbol{K}}.\Delta {\boldsymbol{R}})}){P}_{{c}_{q}}({\boldsymbol{K}}){d}^{d}{\boldsymbol{K}}\ldots {d}^{d}{\boldsymbol{K}}$$

Following a procedure similar to that used in the derivation of spectra^37^ based predictability limits, the scaling exponent of ${P}_{{c}_{q}}$ is derived, as shown here. From the generalized scaling law Eq. (1), it follows that

15 $$\langle {(\Delta f(\Delta {\boldsymbol{R}}))}^{q}\rangle ={[\kern-2pt[ {\boldsymbol{\Delta }}{\boldsymbol{R}}]\kern-2pt] }^{\xi (q)};\xi (q)=qH-K(q\eta )+qK(\eta ),$$

Using the anisotropic functional scale equation^30^ (i.e., Eq. 2) in Eq. (15) gives

16 $$\langle {(\Delta f({T}_{\lambda }\Delta {\boldsymbol{R}}))}^{q}\rangle ={\lambda }^{-\xi (q)}\,\langle {(\Delta f(\Delta {\boldsymbol{R}}))}^{q}\rangle ,$$

whereas using Eq. (14) in Eq. (16) results in

17 $$\begin{array}{rcl}\langle {(\Delta f(\Delta {\boldsymbol{R}}))}^{q}\rangle  & = & {\lambda }^{\xi (q)}\langle \,{(\Delta f({T}_{\lambda }\Delta {\boldsymbol{R}}))}^{q}\rangle \\  & = & {\lambda }^{\xi (q)}{\lambda }^{{D}_{el}}\int \ldots {\int }_{{D}^{(q-1)d}}(\mathop{\sum }\limits_{n=0}^{q}{(-1)}^{n}(\begin{array}{c}q\\ n\end{array}){e}^{i(q-n)(\tilde{{T}_{\lambda }}{\boldsymbol{K}}.{T}_{\lambda }\Delta {\boldsymbol{R}})}){P}_{{c}_{q}}(\tilde{{T}_{\lambda }}{\boldsymbol{K}}){d}^{d}{\boldsymbol{K}}\ldots {d}^{d}{\boldsymbol{K}},\end{array}$$

where $\tilde{{T}_{\lambda }}={\lambda }^{\tilde{G}}$ is the Fourier space scaling operator with the Fourier space generator matrix $\tilde{G}$, ***K*** = (*k*, ω) is the Fourier space wave vector and *D*_*el*_ is the elliptical space-time dimension. The scale invariance of the scalar product ***K***.**Δ*R*** implies that $\tilde{G}={G}^{{\rm{T}}}$. From Eq. (17)

18 $$\begin{array}{c}\langle {(\Delta f(\Delta {\boldsymbol{R}}))}^{q}\rangle \\ \,=\,{\lambda }^{{D}_{el}+\xi (q)}\,\int \ldots {\int }_{{D}^{(q-1)d}}(\mathop{\sum }\limits_{n=0}^{q}{(-1)}^{n}(\begin{array}{c}q\\ n\end{array}){e}^{i(q-n)({\boldsymbol{K}}.\Delta {\boldsymbol{R}})}){P}_{{c}_{q}}(\tilde{{T}_{\lambda }}{\boldsymbol{K}}){d}^{d}{\boldsymbol{K}}\ldots {d}^{d}{\boldsymbol{K}},\end{array}$$

where $\tilde{{T}_{\lambda }}{\boldsymbol{K}}.\,{T}_{\lambda }\Delta {\boldsymbol{R}}$ has been replaced by ${\boldsymbol{K}}.\,\Delta {\boldsymbol{R}}$ (since $\Delta {\boldsymbol{R}}{\boldsymbol{^{\prime} }}={T}_{\lambda }\Delta {\boldsymbol{R}}$ and ${\boldsymbol{K}}{\boldsymbol{^{\prime} }}=\tilde{{T}_{\lambda }}{\boldsymbol{K}}$ and scale invariance of the scalar product means that ${\boldsymbol{K}}{\boldsymbol{^{\prime} }}.\,\Delta {\boldsymbol{R}}{\boldsymbol{^{\prime} }}={\boldsymbol{K}}.\,\Delta {\boldsymbol{R}}$). Equation (18) when compared with Eq. (14), results in

19 $${P}_{{c}_{q}}(\tilde{{T}_{\lambda }}{\boldsymbol{K}})={\lambda }^{-{D}_{el}-\xi (q)}{P}_{{c}_{q}}({\boldsymbol{K}}).$$

The general solution of this functional equation (Eq. (19)) [found by adopting a procedure following chapter 7 of Lovejoy and Schertzer^30^, similar to that subsequently used in Appendix A of Ramanathan *et al*.^43^, and using it along with the anisotropic functional scale equation] is ${P}_{{c}_{q}}({\boldsymbol{K}})\propto {{[\kern-2pt[ {\boldsymbol{K}}]\kern-2pt] }_{FS}}^{-{D}_{el}-\xi (q)}$, so that the scaling exponent of ${P}_{{c}_{q}}$ is $(-{D}_{el}-\xi (q))$. The *q*-th order correlated polyspectrum is therefore ${E}_{{c}_{q}}({\boldsymbol{K}})\propto {{[\kern-2pt[ {\boldsymbol{K}}]\kern-2pt] }_{FS}}^{-1+{D}_{el}}{P}_{{c}_{q}}({\boldsymbol{K}})\propto {{[\kern-2pt[ {\boldsymbol{K}}]\kern-2pt] }_{FS}}^{-{\beta }_{q}},$ where ${\beta }_{q}=1+\xi (q)=1+qH-K(q\eta )+qK(\eta )$ is the polyspectral exponent (*H* and *η* are the conservation or fluctuation exponent and exponent of the conservative turbulent flux respectively, whereas $K(q)=\frac{{C}_{1}}{\alpha -1}({q}^{\alpha }-q)$ is the moment scaling function [not to be confused with the Fourier space wave vector ***K*** = (*k*, *ω*)]). By repeating the above steps but for spatial scaling laws instead of space-time scaling laws and assuming statistical stationarity, it follows that the total polyspectrum ${E}_{{T}_{q}}\propto {|{\boldsymbol{k}}|}^{-{\beta }_{q}}$.

## Supplementary information

Supplementary Information
