# Supplementary material for: Higher-order statistics based multifractal predictability measures for anisotropic turbulence and the theoretical limits of aviation weather forecasting
Source: Sci Rep. 2019 Dec 27;9:19829. doi: 10.1038/s41598-019-56304-2 (PMC6934490; doi:10.1038/s41598-019-56304-2)
Supplement: Supplementary file 1 — Supplementary Information [file 41598_2019_56304_MOESM1_ESM.pdf]

Supplementary Information File

Manuscript Title: Higher-order statistics based multifractal predictability measures for anisotropic turbulence and the theoretical limits of aviation weather forecasting

Author list: 1) Arun Ramanathan[1]

2) ANV Satyanarayana[12]

[1]*Centre for Oceans, Rivers, Atmosphere and Land sciences (CORAL),*

Indian Institute of Technology Kharagpur (IIT KGP), India

[2]Correspondence to [anvsatya@coral.iitkgp.ac.in]

Supplementary Information:

Space-time scale functions

Atmospheric space-time scaling laws are of the general form[1]

$$\Delta f(\boldsymbol{\Delta R}) = |f(\boldsymbol{R} + \Delta \boldsymbol{R}) - f(\boldsymbol{R})| = \varphi \, [\![\boldsymbol{\Delta R}]\!]^H \; ; \; \varphi = \frac{\varepsilon^\eta}{\langle \varepsilon^\eta \rangle} \qquad \text{(S1)}$$

where $\Delta f(\boldsymbol{\Delta R})$ is the fluctuation of the nonconservative turbulent field f across a space-time vector displacement (lag) or scale $\boldsymbol{\Delta R} = (\Delta x, \Delta y, \Delta z, \Delta t)$, $\boldsymbol{R} = (\boldsymbol{r}, t)$ is the space-time position vector, $\boldsymbol{r} = (x, y, z)$ is the spatial position vector and the angular bracket denotes ensemble averaging. The scaling exponent H is the order of fractional integration $(H > 0)$ or differentiation $(H < 0)$, whereas φ is the normalized η^{th} power of some conservative turbulent flux field ε. In other words, H is the conservation or fluctuation exponent, whereas η is the exponent of the conservative turbulent flux. The anisotropic

space-time scale function $[\![\boldsymbol{R}]\!]$ is the general solution of the anisotropic functional scale equation

$$[\![T_\lambda \boldsymbol{R}]\!] = \lambda^{-1}[\![\boldsymbol{R}]\!]\ ; T_\lambda = \lambda^{-G_{st}}$$

$$G_{st} = \begin{bmatrix} G_s & 0 \\ 0 & H_t \end{bmatrix};\ G_s = \begin{bmatrix} d-c & f+e & 0 \\ f-e & d+c & 0 \\ 0 & 0 & H_z \end{bmatrix}, \tag{S2}$$

where T_λ is the scale changing (transformation) operator, G_{st} and G_s are the space-time and spatial generator matrices, H_t is the dynamic exponent or the space-time anisotropy parameter, H_z is the vertical stratification exponent, λ is the scale ratio and c, d, e, f are the generalized scale invariance (GSI) parameters[2]. The spatial elliptical dimension $D_{el,s}$ equals the trace of the matrix G_s, whereas the space-time elliptical dimension $D_{el,st}$ is the trace of the matrix G_{st}. Following Marsan et al.[3,4] the canonical space-time scale function in real (physical) space can be taken as

$$[\![(\Delta\boldsymbol{r}, \Delta t)]\!] = L\big((|\Delta\boldsymbol{r}|/L)^\sigma + (|\Delta t|/T)^{\sigma/H_t}\big)^{1/\sigma};$$

$$|\Delta\boldsymbol{r}| = (|\Delta x|^\sigma + |\Delta y|^\sigma + |\Delta z|^\sigma)^{1/\sigma};\ \sigma > 0\,, \tag{S3}$$

where $|\Delta\boldsymbol{r}|$ is the isotropic spatial scale function, L is the integral length scale (usually taken as the size of the largest eddy) and T is the eddy turnover time corresponding to L. The main difference between real space and fourier space scale functions is that they are symmetric with respect to different generators: G_s and G_s^{T} (this superscript 'T' indicates the transpose of a matrix, not to be confused with the integral time scale T or the scale transformation operator T_λ). For spatially isotropic, self-affine cases or GSI cases with no differential rotation of structures ($e = 0$), G_s is symmetric so that $G_s = G_s^{\mathrm{T}}$. The Fourier

space scale function (indicated by the subscript FS) corresponding to the real space scale function can therefore be taken as

$$\llbracket(\boldsymbol{k},\omega)\rrbracket_{\mathrm{FS}} = K_i\left((|\boldsymbol{k}|/K_i)^{\sigma} + (|\omega|/\Omega_i)^{\sigma/H_t}\right)^{1/\sigma};$$
$$|\boldsymbol{k}| = \left(|k_x|^{\sigma} + \left|k_y\right|^{\sigma} + |k_z|^{\sigma}\right)^{1/\sigma};\ \sigma > 0\ ; K_i = \frac{2\pi}{L}\ ; \Omega_i = \frac{2\pi}{T} \quad \text{(S4)}$$

where $|\boldsymbol{k}|$ is the isotropic spatial scale function in Fourier space, $\boldsymbol{k} = (k_x, k_y, k_z)$ is the wavevector, K_i and Ω_i are the (angular) wavenumber and (angular) frequency corresponding to the integral length and time scales (L and T) respectively. Even though this study prefers the theoretical physics convention of using angular frequency ω and angular wavenumber k (although the word angular is often dropped in the manuscript for convenience) in the Fourier space instead of the alternate spectroscopy convention of using frequency and spectroscopic wavenumber, both the conventions result in the same final outcome as long as they are used consistentently.

Semi-Fourier Space scale functions

Taking into consideration the scaling anisotropy between space and time (based on the Kolmogorov-Obukhov law[5,6]) suggested by earlier studies[7,8], $|\omega| \propto |k|^{H_t}$ can be squared on both sides and non-dimensionalized using Ω_i and K_i to get $\left(\frac{|\omega|}{\Omega_i}\right)^{2/H_t} \propto \left(\frac{|\boldsymbol{k}|}{K_i}\right)^2$. Since a spatial-scale dependent but position independent expression for predictability limit is what is needed, it is advantageous to work in semi-Fourier space, fully exploiting this spatial position independence property. The purpose of squaring, is to obtain an expression for $|\omega|^{1/H_t}$ in terms of $|\Delta t|$, that can be raised to power σ and used in Eq. (S4)

to replace the $|\omega|^{\sigma/H_t}$ term. To do this $\left(\frac{|\omega|}{\Omega_i}\right)^{2/H_t} \propto \left(\frac{|\boldsymbol{k}|}{K_i}\right)^2$ is rewritten as $\left(\frac{|\omega|}{\Omega_i}\right)^{\frac{1}{H_t}} \propto \frac{\left(\frac{|k|}{K_i}\right)^2}{\left(\frac{|\omega|}{\Omega_i}\right)^{\frac{1}{H_t}}}$,

which when using $|\omega| = 2\pi/|\Delta t|$ only on the right hand side becomes

$$|\omega|^{1/H_t} \propto \frac{(\Omega_i)^{2/H_t}\,|\Delta t|^{1/H_t}\,|\boldsymbol{k}|^2}{(2\pi)^{1/H_t}\,(K_i)^2}, \tag{S5}$$

which when raised to power σ and substituted in Eq. (S4) as discussed above gives

$$[\![(\boldsymbol{k},\omega)]\!]_{\mathrm{FS}} \propto \left(|\boldsymbol{k}|^\sigma + \frac{(L)^\sigma \left(\frac{|\Delta t|}{T}\right)^{\frac{\sigma}{H_t}} |\boldsymbol{k}|^{2\sigma}}{(2\pi)^\sigma}\right)^{\frac{1}{\sigma}} = [\![(\boldsymbol{k},\Delta t)]\!]_{\mathrm{SFS}}\,; \tag{S6}$$

where $\sigma > 0\,; K_i = \frac{2\pi}{L}\,; \Omega_i = \frac{2\pi}{T}$ as usual and the subscript ‘SFS’ denotes the semi-Fourier space. Using $E_{c_q}(\boldsymbol{k},\omega) \propto [\![(\boldsymbol{k},\omega)]\!]_{FS}{}^{-\beta_q}$ from Methods section with Eq. (S6) implies that $E_{c_q}(\boldsymbol{k},\Delta t) \propto [\![(\boldsymbol{k},\Delta t)]\!]_{SFS}{}^{-\beta_q}$.

Supplementary References:

1. Lovejoy, S. & Schertzer, D. *The Weather and Climate: Emergent Laws and Multifractal Cascades*. (Cambridge University Press, 2013). doi:10.1017/CBO9781139093811
2. Schertzer, D. & Lovejoy, S. Multifractals, Generalized Scale Invariance and Complexity In Geophysics. *Int. J. Bifurc. Chaos* **21**, 3417–3456 (2011).
3. Marsan, D., Schertzer, D. & Lovejoy, S. Causal space-time multifractal processes: Predictability and forecasting of rain fields. *J. Geophys. Res. Atmos.* **101**, 26333–26346 (1996).
4. Marsan, D., Schertzer, D. & Lovejoy, S. Predictability of multifractal processes: the case of turbulence. *Fractals Chaos Chem. Eng. CFIC 96, Eds. M. Giona, G. Biardi, World Sci.* 421–433 (1997).

5. Obukhov, A. M. On the Energy Distribution in the Spectrum of a Turbulent Flow (in Russian). *Dokl. Akad. Nauk SSSR* (1941). doi:http://dx.doi.org/10.1016/B978-012370615-7/50053-6
6. Kolmogorov, A. N. The Local Structure of Turbulence in Incompressible Viscous Fluid for Very Large Reynolds Numbers. *Proc. R. Soc. A Math. Phys. Eng. Sci.* (1991). doi:10.1098/rspa.1991.0075
7. Schertzer, D. *et al.* New developments and old questions in multifractal cloud modeling, satellite retrievals and anomalous absorption. in *7th ARM Science Team Meeting* (1997).
8. Schertzer, D. & Lovejoy, S. Uncertainty and predictability in geophysics: Chaos and multifractal insights. in *Geophysical Monograph Series* **150**, 317–334 (2004).
